# Hospital and Institutional News

**Published:** 1912-10-26

**Authors:** 


### HOSPITAL AND INSTITUTIONAL NEWS.

### THE STATUS OF THE WORKHOUSE MEDICAL OFFICER.

We publish elsewhere in this issue an illuminating interview with Dr. C. T. Parsons, medical superintendent of Fulham Poor-Law Infirmary, on the status of the workhouse medical officer. Dr. Parsons' wide experience fully entitles him to speak on the subject which the recently issued Draft Poor-Law Order has brought into the limelight once more. In some carefully-considered criticisms the present lot of the workhouse medical officer is described, and the argument, with many illustrations of weight, is strongly enforced for the complete autonomy of the medical officer in the sick-wards of workhouses wherever these happen to be situated. Dr. Parsons, moreover, proceeds to show that the unattractive nature of the Indoor Service is not merely the result of under-payment and divided responsibility, and that a far larger problem must be tackled if the Service is to produce the results of which it is capable. He outlines the reforms which would follow if the administrative areas were enlarged, and if, in the case of London, the thirty-one separate Boards of Guardians were linked up under one administration. To the details of this scheme our readers will turn with intense interest; and, meantime, it must not be forgotten that if the status of workhouse medical officers is to be improved, it is now or never for them to press their demands on the Departmental Committee. As Dr. Parsons observes, if the Draft Order be confirmed medical officers will have a long time to wait before any opportunity will occur for further amendment.

### STARTING FOR THE FRONT.

Surgeon-General Bourke left London on Sunday night in charge of the first surgical unit which the British Red Cross Society has raised for service in the Bulgarian-Turkish War. Thanks to the energy of the Medical Relief Sub-Committee of the Society, the unit, which consists of three dressers and twelve orderlies, has been chosen, equipped, and sent off in four days. The surgeons were Dr. Anthony Bradford, late resident medical officer at St. Thomas's Hospital Home for Paying Patients; Dr. F. Goldsmith, of Adelaide, Australia; and Surgeon-Captain Martin-Leake, V.C. The unit was expected to arrive at Cettigne, Montenegro, on Thursday last. A notable send-off was given on the platform at Victoria by Sir Frederick and Lady Treves, the former being chairman of the Medical Relief Sub-Committee, Sir Anthony Bowlby, Mr. A. E. Ridsdale, chairman of the executive, and Mr. G. H. Makins, C.B., who, it will be remembered, acted as consulting surgeon to the South African Field Force in 1899 to 1900. In addition, through the generosity of Sir Ernest Cassel, three further units left on Friday. To how great an extent the Society's services are appreciated by the combatants may be judged from the fact that, in spite of the poor results achieved by the appeal on behalf of the Balkan Relief Fund, the Society hopes this week to comply with the urgent requests of Greece for surgical assistance. It may be expected, therefore, that if funds permit further units will be despatched, and after we go to press this week it is possible that arrangements for the enrolling of one or two further units may have been completed. All the workers on the units, we understand, are paid, the dressers receiving 40s. per week and the orderlies 25s. to 30s. On November 7 a subscription ball will be held at the Mansion House on behalf of the British Red Cross Balkan Fund.

### THE SWEATING OF POOR-LAW MEDICAL MEN.

The recent appointment of Dr. Cohen as senior tuberculosis medical officer to the Hull Corporation at a salary of £500 a year has led to a vacancy through his resignation of the post of medical officer to the workhouse. The salary of the latter post is only £225, which Dr. Cavanagh, one of the Guardians, stigmatised as insufficient, and was supported by Mr. Booth, who pointed out that the appointment was "by no means a plum." These gentlemen moved and seconded a resolution to increase the remuneration to £250. It is a relief to find Guardians, or at any rate individual members, beginning to awake to the fact that the Poor-Law Medical Service, in its indoor posts at any rate, is very badly underpaid, and that unless the Service be made more attractive it will become quite impossible to attract the best men. The disastrous system which so generally compels the workhouse medical officer to provide drugs out of his salary is another feature which makes the salaries offered even worse in fact than they seem on paper, and on paper they are certainly uninviting enough. Mr. Booth, therefore, is to be congratulated on his bold statement of fact that even £250 was too little for the amount of work required, and we cordially hope that his influence will permeate the Board until it frankly recognises, and so sets an example to Boards elsewhere, that the policy of sweating workhouse medical officers must be stopped if a premium is not to be placed on scamped work and on inferior candidates.

### THE FINAL OFFER TO THE DOCTORS.

By the time that this issue is in the hands of our readers the final terms to be offered to the medical profession in order to secure their cooperation in working the Insurance Act will probably be known. A Cabinet Meeting to discuss them was held on Tuesday, at which it was believed the decision was arrived at to offer a lump sum to increase the fees. On Wednesday Mr. Lloyd George met the Advisory Committee; as we go to press it is expected that if this meeting proves satisfactory he will make a categorical reply to Sir Philip Magnus' question in the House. Interest attaches to the Government's solution of the problem presented by the difference in cost of medical service in town and country, to the question of mileage allowance for practitioners in rural districts, and to the amount to be allowed for the provision of drugs. The alternative to the rejection of this " revised " offer is believed to be a State Medical Service, which, however, is understood not to recommend itself to the Ministry. (See page 89, " Payment of Doctors Abroad," and p. 102.)

### THE NEW MURRAY HOSPITAL NEAR NEWCASTLE.

Certainly one of the most individual, if not the most striking, of the King Edward memorials will be the Richard Murray Commemoration Hospital, the foundation-stone of which was laid at Blackhill, near Newcastle, last week. On the death of King Edward, Mr. Richard Murray, who had fought his way up from errand boy and shop assistant to shop proprietor and capitalist, decided to do something for his district by building, equipping, and partly endowing a hospital. His total gifts amount to £57,000, of which £10,000 was deposited for building and £20,000, in the form of shares in the North Eastern Brewery Company, for endowment purposes. The founder's aim is to cure and nurse back through convalescence to health the patients through this institution. The Consett district welcomes the new institution on the ground, it is said, that the demands on the Newcastle Infirmary are so great that though the workers subscribe they cannot be sure of admission without delay. The plans adopted by Mr. J. J. Eltringham, the architect, arrange for two end blocks connected by a long corridor, in which, as stated, an important feature will be the convalescent ward. The ground floor is devoted to male patients, and the upper floor which corresponds to it will be given up to women, with an addition of eight cots for children. The secretary is Mr. A. E. Coldwell, and Mr. John T. Murray is the chairman.

### THE FIRST WOMAN HOSPITAL FOUNDER.

A tablet is about to be placed on the wall of the Edinburgh Hospital for Women and Children to perpetuate the memory and career of Dr. Sophia Jex-Blake, the founder of the institution. The tablet's inscription, which is engagingly terse and to the point, reminds her successors that to her " large courage, insight, and constancy the admission of women to the profession of medicine in this country was mainly due." The tablet has additional interest from the fact that it is probably the first to record the foundation of a hospital by a woman doctor, who struck a double blow for medicine and for her sex in associating herself with the institutional as well as the scientific side of medicine. No doubt it was the opposition which she and her friends encountered in their attempts to study medicine that led her to found the Edinburgh Hospital for Women and Children, but that fact merely illustrates how the most bitter enemies of a movement often do indirectly as much for it as its best friends, and how, indeed, a pioneer of character and determination uses every obstacle as a lever and aid. The late Sophia Jex-Blake will always have an honoured place among the historic band of hospital founders.

### WOMEN AND THE LUNACY COMMISSIONERS.

An animated discussion arose at a recent meeting of the Standing Committee of the House of Commons, presided over by Sir D. Brynmor Jones, further to consider the Mental Deficiency Bill. The question over which most excitement arose was whether women were desirable as commissioners. One member even went so far as to say that the Government was afraid of the women, and so they " were shoved into every Bill put before the House." Joking apart, however, it is rather late in the day to criticise adversely women in public life, especially as in the case of these commissioners the opinion was expressed that one of them infallibly would be a woman doctor. The Home Secretary eventually suggested that words should be inserted to secure that one of the paid commissioners should be a woman, and one of the unpaid members also. His amendment to this effect was carried, and he then stated that in the first instance he did not propose to appoint more than eleven paid commissioners—that is, he explained, three in addition to the existing eight Lunacy Commissioners, who will form the original Board of Control.

### THE CASE FOR THE SHEFFIELD ROYAL HOSPITAL.

The burden of debt was made the subject of lamentation at the annual meeting of the Sheffield Royal Hospital last week. A deficit of £8,000 as the result of the past two years was complained of, and owing to lack of funds it was stated that the nurses' and servants' home could not be proceeded with. This means that the accommodation for the staff necessary in connection with the new block is wanting, and until it is provided that the new wards in the Princess of Wales' block will have to remain closed. The building fund is the serious item, as the amount due on it is £13,412. Various speakers, including the Lord Mayor, Mr. A. J. Hobson, gave various reasons, legislation among them, for the fact that the income does not keep pace with the expenditure, and the Rev. J. H. McNeal said that the clergy had undertaken to make greater efforts on behalf of the hospital. Examples were also given of the congestion which exists, and of the great pressure on the hospital's accommodation. In short, an air of depression and gloom brooded over the annual meeting. In the face of that gloom we would say this. Notwithstanding the difficulties which are the common heritage of the voluntary hospitals, the history of voluntary hospital finance is the most cheerful financial history of any branch of charity in the kingdom. Again and again a position similar to that of the Sheffield Royal Hospital has arisen and been made by a new effort the excuse for raising a large sum of money. One of the most striking financial resurrections of recent years has been that achieved by King Edward VII.'s Hospital, Cardiff. Let Mr. P. K. Wake, the chairman of the Royal Hospital board, turn either to our former issues or to Cardiff to learn how that great overdraft was paid off. The Sheffield Royal Hospital has such a strong case that it only requires its presentation to be organised and a time limit to be set for the bulk of the money required to be raised. But it needs a special effort. Our one criticism of the annual meeting is that no effort was promulgated. The time between now and Christmas is the time when it should be made.

### HOSPITAL APPEALS.

There is no question that if ability and business method can produce large funds for a voluntary hospital in these days, the authorities of Charing Cross Hospital should soon have at the bank all the money they require. For the last few years an able and persistent appeal has been worked with marked ability and excellent method. The psychological moment for appeals has been chosen, and the points most calculated to tell at the date each letter is written are to be found in the Charing Cross literature. The present week brings us the latest letter of request, which is issued under the authority of the finance committee in the ordinary course, which relies upon its terse recital of the facts for its success, and is not a mere lithographic production bearing the signature of some eminent person. This in our view is good business, and we hope the results, as we think they may do, will prove to be exceptionally good. In this connection we may add that the report of the Charing Cross Hospital issued this year will well repay the closest inspection. It does not contain one dull page, and every page is attractive to the eye and so inviting to the recipient. Everything that thoughtful arrangement, a careful selection of type, knowledge, and, we should even think, an affection for good printing and excellent workmanship can produce may be found within the pages of the report of the Charing Cross Hospital, London, for the year ending December 31, 1911. Mr. Walter Alvey is entitled to grateful recognition and thanks for the excellence of the work to which we here call attention.

### "PAYMENT OF DOCTORS ABROAD."

Under this heading the *Daily News* of the 18th instant has a notice of a book, "Medical Benefit in Germany and Denmark," by Dr. Gibbon. We propose to deal with this book fully in an early issue. The *Daily News* states that this book "makes it clear that the present proposals of the British Government have been made in a liberal spirit considering the terms upon which doctors have worked in the two countries under review." It then proceeds to show, in support of this statement, that the average cost of insured persons to doctors for medical treatment was *only* 4s. 7d. in 1909. Now the scale of payments under Mr. Lloyd George's Bill was 6s., of which not more than 4s. can be available for the payment to the practitioner for medical treatment, seeing that the experience of Germany proves that the balance of 2s. is not enough to defray the cost of drugs. We have here a striking example of the way in which the public may be misled in the political press on matters of fact. We may add that it is a notorious fact that the payment to general practitioners in Germany is so miserably inadequate that the younger and better qualified members of the medical profession in increasing numbers prefer to remain at nominal salaries as resident officers of the hospitals rather than to enter general practice. So far from proving the *Daily News* claim that the present proposals of the British Government to the medical profession have been made in a liberal spirit, they demonstrate on the contrary that the demand of the British practitioners is not only reasonable but indispensably necessary if the public of all classes are to have available in the time of sickness a medical service manned by the best qualified men which the medical profession contains. Thus the *Daily News* states that in Denmark, where the cost of living is infinitely cheaper than in Great Britain, the societies in Copenhagen will in 1915 pay the doctors for medical treatment 5s. per member. British doctors ask 8s. 6d., that is 6s. per insured person for medical treatment and 2s. 6d. for drugs, etc. The medical profession's claim in Great Britain for 6s. per member is only 1s. more than that to be paid in Copenhagen by the friendly societies—that is, for selected lives. In Great Britain the doctors under the National Insurance Act will have to attend all insured persons, not selected lives only, for a payment of 6s. It follows that the rate in Denmark, and the rates asked for by members of the medical profession in this country are, when the cost of living and the question of selected and unselected lives are taken into account, almost identical. What a reflection these facts are upon the conduct of members of the Government and political partisans who have been denouncing English doctors throughout the country as greedy, hungry harpies, when they are in fact honourable gentlemen who appreciate all the facts which the present party in power would not trouble to learn, and with this knowledge prove themselves to be moderate and businesslike in the just proposals they have submitted to Mr. Lloyd George and the Government. The precise accuracy of this view is demonstrated by the concessions Mr. Lloyd George announced in the House of Commons on the 23rd inst.

### A NEW MEDICAL TRAINING SCHOOL.

The opening of the new medical school attached to the Royal Hospital for Diseases of the Chest in City Road was the occasion of an inaugural address by Professor Nietner, of Berlin. He emphasised the importance of the careful organisation of the school medical service in fighting tuberculosis, and said that to the school doctor alone was given the power to prevent latent tuberculosis from developing into active disease in the children placed under his supervision. It is with the practical reforms which follow from these dicta that the new medical training school is concerned. A training in the newly-equipped out-patient department will be given to medical officers of tuberculosis dispensaries and sanatoria, and particularly to general practitioners, and also to nurses. Insistence on its importance has been made in Germany, where it has become generally recognised that domiciliary work, such as that connected with dispensaries, necessitates efficient training, in particular with regard to the collection and interpretation of statistics and data. Without it the right sort of information is not forthcoming. There is no doubt that on this side of the Channel in many respects we are behind the organised co-operation to be found in Germany, but perhaps here again we shall reap some reward of our dilatoriness by avoiding the mistakes incidental to the tentative beginnings of foreign pioneers.

### AN INQUIRY INTO INFANT MORTALITY.

At a lecture last week on the "Social Application of Eugenics" Mr. E. J. Lidbetter suggested an inquiry on a large scale into the causes of infant mortality. It is not a new suggestion and might well prove productive, provided that it is set about in the right way. What the right way is has been laid down authoritatively of late by Havelock Ellis, whose masterly chapters on infant mortality and its direct relation to a falling birth-rate were reviewed in these columns some weeks ago. The main point is to study the question of the birth-rate and infantile mortality rates conjunctively and not apart if the conclusions are not to be vitiated from the outset. As there exists such an inordinate amount of confusion and unfounded alarm in the public mind on the question we may hope that the lecturer's suggestion may be adopted by a reliable authority, whose competence may be judged by his recognition of the conjoint nature of the inquiry.

### A CURIOSITY IN ADMINISTRATIVE BLOCKS.

A new administrative block, with porter's lodge, is proposed by the Acton Urban District Council for the isolation hospital in Wales Farm Road. For pressing this the Medical Officer of Health must be given his due. The present administrative block is an old converted mansion, which, in Dr. D. J. Thomas's opinion, is utterly unsuitable for its purpose, inasmuch as most of the bedrooms have sloping roofs of a maximum height of 6 feet 6 inches, tapering down to less than 3 feet. Exigencies of space have compelled the Council to lease and furnish a house closely adjoining the hospital, in order to provide sleeping accommodation for the nursing staff, but the Medical Officer is strongly opposed to such a procedure, except quite as a temporary expedient. He points out that the present conditions of the administrative block not only make administration difficult, but also expensive. At the present day, in fact, such conditions are becoming a curiosity.

### SOUTHWARK GUARDIANS AND GUY'S HOSPITAL.

We have had occasion before now to remark on the antagonism which exists between the Southwark Board of Guardians and Guy's Hospital, and we regret to notice that the trouble has broken out again. We previously expressed the opinion that the Southwark Guardians have been remiss about providing proper infirmary accommodation for those who are entitled to seek it, and that in consequence of this a good many poor people seek treatment at Guy's Hospital who are not proper voluntary hospital cases at all. Confronted with a case of chronic disease which has reached a moribund stage, or with an acute case for which no bed is available, the custom at Guy's would appear to be to put the patient in a cab and send him to the relieving officer, should he be a resident in Southwark and unable to provide medical attendance for himself. This policy has, it may be supposed, been adopted in order to bring home to the Guardians what their responsibilities are; but it has the disadvantage of laying the hospital open to hostile comment every now and then. Three such cases have occurred within the last few days; and if the account of what happened which has appeared in the press correctly represents the facts, we can only say that in one case a grave error was committed, and that in the other two the action taken was very injudicious. We have the right to suppose that the Board of Guardians and the governors of Guy's include level-headed men of the world, who have only to meet in a friendly discussion to understand each other's points of view and to take such action as shall end these bickerings about the sick poor of Southwark, who meanwhile have to suffer for the quarrel.

### THE VICE-PRESIDENCY OF THE ROYAL COLLEGE OF SURGEONS.

The death of Mr. Clinton Dent created a vacancy for a vice-president of the Royal College of Surgeons, and the Council of the College has elected Mr. Edmund Owen to this honourable position. Mr. Owen's long connection with St. Mary's Hospital is familiar to the hospital world in London, and his many other activities—in connection with the "Encyclopædia Britannica," for example—are a sufficient guarantee of a broad outlook and a worldly sagacity such as the profession rightly expects of an officer at the College of Surgeons. Mr. Owen has a fine presence, a kindly humour, and a reputation for common sense as well as surgery. To the younger generation he is well known as a merciful and indefatigable examiner; and his popularity is not confined to St. Mary's. We congratulate him on his well-deserved promotion.

### THE BRITISH HOSPITALS ASSOCIATION.

A meeting of the Council of the British Hospitals Association was held at the Hospital for Sick Children, Great Ormond Street, London, on October 16. Dr. Mackintosh, chairman of the Council, presided, and there was a large attendance of members, including representatives from Edinburgh, Manchester, Birmingham, Sheffield, Leamington, and Wellingborough. An executive committee and a finance committee were appointed. Mr. Stewart Johnson, the honorary treasurer, presented a financial statement, and announced that, with few exceptions, every important hospital was represented in the Association, but it was very desirable that every hospital or infirmary in the country should join. Mr. Conrad Thies, the honorary secretary, stated that the full report of the proceedings of the third annual Conference, recently held in Birmingham, was in the hands of the printers, and would shortly be issued to the members. The most important business under consideration by the Council referred to the next annual Conference. The majority of the members present were of the opinion that it should be held in one of the provincial cities—Bristol and Oxford were both suggested—and it was generally agreed that the Conference should take place in either June or July. It was left to the executive committee to make the necessary arrangements. The question of the unwillingness of the honorary medical staffs to treat in-patients, insured persons, which had already been expressed in some instances, was discussed, and it was agreed that, pending the negotiations between the Chancellor of the Exchequer and the medical profession, it was not expedient for the Council, at the present time, to formulate any resolutions upon this difficult question, which would, however, continue to receive careful consideration by the executive committee.

### HOSPITALS AND THE COMPULSORILY INSURED.

In his paper on Hospitals and the State, published in The Hospital of September 28, page 678, Sir William Collins wrote: "The experience in Germany of the refusal of the compulsorily insured, when in hospital, to be regarded as 'clinical material' is also significant." Professor Kerschensheimer, writing under date of 10th instant, referring to this statement, says: "Your information that the members of the Kasse" (friendly society, *i.e.*, the compulsorily insured) "are a class of patients apart, are not used for teaching purposes," and that no post-mortems are made is wrong. These patients are kept like all the others (*i.e.*, in the Stadt or municipal hospitals); all patients have the right to refuse clinical investigation by students, and, of course, the Kasse patients also. It is very seldom that any patient refuses clinical investigation. With the sections (post-mortems) it is the same. The treatment of Kasse patients is in no way other than that of all patients in the "Commun-saalen" or ordinary wards. Seeing that no patients are admitted to a teaching hospital, except they agree to clinical investigation by students, it is clear that in Stadt and municipal hospitals in Germany the compulsorily insured are not kept as a class of patients apart, and that they are used for teaching purposes and are subject to autopsies in the pathological department in the same way as all other patients are subject, except a particular patient refuses to submit to clinical investigation by students. Such a refusal by a patient on seeking admission to a teaching hospital in Germany would prevent his admission as an in-patient. In the municipal or town hospitals every patient has the right to refuse to submit, but if exercised in the case of a municipal hospital he may or may not be admitted as an in-patient.

### PROGRESSIVE DEVELOPMENTS AT LEICESTER INFIRMARY.

In his quarterly report Mr. Harry Johnson, secretary and house governor of the Leicester Royal Infirmary, referred to the success which has attended the new massage, electrical, and Swedish exercises department. No fewer than 150 patients per week have received treatment, and, though the treatment itself is of considerable length, the board learns that excellent results have been obtained. The other special departments of the hospital—the ophthalmic, ear, and throat—have received 403 out-patients, who have come from all parts of Leicestershire and Rutland as a result of the systematic medical inspection of school children. It is hoped, however, that the cliniques which the Borough Education Committee is adopting will relieve the hospital of this additional and very heavy work. This is the age of special departments, even though it must be admitted that orthopædics in this country only now are beginning to receive their due in this respect. If for no other reason the Royal Infirmary at Leicester deserves the support it is asking for at this moment, and we hope that the organisers of Hospital Sunday will not have worked in vain.

### THE IMPORTATION OF AMERICAN REMEDIES.

The first witness to appear before the Select Committee on Proprietary Medicines when the inquiry was resumed was Dr. Norman Walker, treasurer of the Royal College of Physicians, Edinburgh, and lecturer on skin diseases at Edinburgh University. His evidence was illustrated by means of a number of plaster casts showing the effects produced on the skin by various drugs and advertised preparations. He admitted at the outset that these effects were sometimes caused by drugs prescribed by physicians, but it is obvious that the case of a patient who is under the care of a medical man, who can observe the effects of his treatment, is totally different from the case of a person who relies upon a proprietary compound of unknown composition. His suggestions for dealing with the "patent medicine" evil were not at all drastic; the important thing was that the names of the active ingredients, rather than their quantities, should be stated. His belief, in common with that of previous witnesses, was that the effect of the Insurance Act will be to diminish self-medication, because the patient will have the right to go to a doctor and get a prescription from him; he would be very foolish if he did not go to a doctor under these circumstances. Another witness was Mr. G. S. Paternoster, assistant editor of *Truth*, who said that there was not a day or a post which did not bring some inquiry or complaint with regard to some secret nostrum or other. He estimated that as much as £400,000 a year was spent in England on imported American remedies, and stated that the cost of catching the customer was more than the cost of the article sold. He also mentioned the interesting fact that there was a Central Exchange at which lists of people who had answered advertisements for remedies could be purchased by the vendors of other remedies. *Truth* has done such valuable work in exposing the evils of the secret medicine traffic while other lay papers have encouraged it, and has collected such a mass of information relating to the traffic, that the continuation of Mr. Paternoster's evidence promises disclosures which should be of great assistance to the Committee.

### EXTENSION OF THE BRITISH HOME FOR INCURABLES.

Last Saturday the foundation-stone of the new Queen Alexandra Wing of the British Home and Hospital for Incurables, Streatham, was laid by Lord Strathcona, who read a telegram from Queen Alexandra thanking the subscribers and expressing her interest in "the valuable and useful addition"

to the institution. The Chairman of the Board read an address to Lord Strathcona, in which the committee's desire to raise £30,000 was explained. It appears that £18,000 has been subscribed, but that the new wing will cost rather more than half of this sum, while a large portion of the amounts received have been given for purposes apart from building, such as endowment. To complete the building fund some £8,000 is required. It is much hoped, as the Dean of Westminster reminded those present, that next year when the wing is finished it may be opened by Queen Alexandra herself.

### THE LATE DR. ANDREW DUNCAN.

By the death of Lieut.-Colonel Andrew Duncan, formerly of the Indian Medical Service, the Seamen's Hospital loses a physician and tropical medicine a gifted exponent. Dr. Duncan took high honours in his student days, and soon after he joined the Service he was severely wounded at the battle of Charasiah in the Afghan campaign. It was, however, after settling in London again that he made his name known in this country; although he did little, if any, actual research in tropical medicine, his wide clinical experience made his services invaluable to the Seamen's Hospital Society. Dr. Duncan was both F.R.C.P. and F.R.C.S., and he enjoyed the rare distinction of having been medical registrar at one London Hospital (Charing Cross), surgical registrar at another (King's College), and pathologist at a third (St. Mary's). He also lectured in later days on tropical medicine at Westminster Hospital, as well as at the London School of Tropical Medicine, and was physician to the Westminster General Dispensary and to the Western General Dispensary.

### DOCTORS' FEES AND MATERNITY BENEFIT.

The letter sent by the Joint Committee of the National Health Commissioners to clerks of Boards of Guardians during the past week asking for their views on the existing practice of payment of fees to practitioners when summoned by certified midwives to attend confinements under the Midwives Act of 1902 has not escaped, we are glad to see, the notice of the Poor-Law Medical Officers' Association. Its Secretary, Major Greenwood, has at once reminded the public that in many cases Boards of Guardians have refused to pay any fee at all, and that even where a fee has been paid it has been one which nearly always compares unfavourably with the £2 fixed in Article 183 of the Consolidated General Order of July 1847, to be paid where the district medical officer is required to give assistance in cases of special difficulty. Major Greenwood also remarks on the experience of the Boards of Guardians being asked for, since both Poor-Law Reports "severely commented on the general inadequancy of the payment in the Poor-Law Medical Service." He concludes as follows: "As the maternity benefit is 30s., and £2 the fee ordered by the Poor-Law Orders to be given to a district medical officer when summoned to assist a midwife, the Insurance Commissioners will find it exceedingly difficult to frame regulations whereby an adequate fee can be paid." The cynic might suggest that that is why, perhaps, the experience of the Guardians has been sought upon the subject.

### VOLUNTEERS AND FREE GIFTS FOR THE WAR.

That the war in the East has produced the usual crop of candidates for admission to the ranks of at least one of the combatants is apparent from a notice issued by the Ottoman Embassy. It states that the Ottoman Embassy is very grateful for the considerable number of applications received from all parts of the British Isles for volunteer service in the Ottoman Army, thanks very warmly, in the name of its Government and of its compatriots, those applicants, and informs them that as the Imperial Government has not yet taken a decision concerning the admission of foreigners in the Ottoman Army, the Embassy regrets to be unable to comply with their demands. The Embassy at the same time "begs to acknowledge receipt of the numerous offers from ladies and gentlemen in the British Isles of sending ambulance materials, such as medicines, bandages, etc., to the Red Crescent hospitals in Constantinople, and to tender in the name of its Government and of its compatriots its very sincere thanks, and to state that their kind offers are thankfully accepted by the Imperial Government and that the materials should be addressed to the President of the Ottoman Red Crescent Society in Constantinople."

### THIS WEEK'S DRUG MARKET.

Up to the present there has been no further advance in the price of opium, but the general opinion is that the lack of means of transport, owing to the hostilities in the Balkans, will lead to higher prices. Manufacturers of acetic acid and acetone have been obliged to raise their quotations for these products owing to the increased demand for acetone required for the manufacture of explosives. Acetate of lime, from which acetic acid and acetone are produced, has been in short supply for some months, and the scarcity has been accentuated by the demand for explosives occasioned by the troubles in the Near East. Acetone is used in the production of a number of medicinal preparations, but it remains to be seen whether the cost of these will be raised in consequence of the higher quotation for acetone; it is probable, however, that the value of such preparations will not be affected. It is not improbable that the hostilities in the Balkans may have an effect on the price of glycerine, which is used in large quantities in the production of dynamite; refined glycerine, however, is not likely to be affected to the same extent. Cocaine hydrochloride is 4d. per ounce dearer. Menthol continues to advance in price. Cod-liver oil is quiet and the price tendency seems to be downwards rather than upwards. On the whole, business has been somewhat quieter during the week.

